# Validation of a Brief Form of the Self-Administered Multidimensional Prognostic Index: The SELFY-BRIEF-MPI Project

**DOI:** 10.3390/jcm12186026

**Published:** 2023-09-18

**Authors:** Wanda Morganti, Nicola Veronese, Marina Barbagelata, Alberto Castagna, Carlo Custodero, Luisa Solimando, Marianna Ilarj Burgio, Sofia Elena Montana Lampo, Emanuele Seminerio, Gianluca Puleo, Barbara Senesi, Lisa Cammalleri, Giovanni Ruotolo, Carlo Sabbà, Mario Barbagallo, Alberto Pilotto

**Affiliations:** 1 Department of Geriatric Care, Orthogeriatrics and Rehabilitation, Ente Ospedaliero Galliera Hospital, 16128 Genoa, Italy; wanda.morganti@galliera.it (W.M.); marina.barbagelata@galliera.it (M.B.); emanuele.seminerio@galliera.it (E.S.); gianluca.puleo@galliera.it (G.P.); lisa.cammalleri@galliera.it (L.C.);; 2 Geriatric Unit, Department of Internal Medicine and Geriatrics, University of Palermo, 90127 Palermo, Italy; luisa.solimando@community.unipa.it (L.S.); mariannailarj.burgio@community.unipa.it (M.I.B.); sofiaelena.montanalampo@community.unipa.it (S.E.M.L.); mario.barbagallo@unipa.it (M.B.); 3 Primary Care Department, Health District of Soverato, Catanzaro Provincial Health Unit, 88068 Soverato, Italy; alberto.castagna@materdominiaou.it; 4 Department of Interdisciplinary Medicine, “Aldo Moro” University of Bari, 70124 Bari, Italy; carlo.custodero@uniba.it; 5 Geriatric Medicine Department, Azienda Sanitario Ospedaliero “Renato Dulbecco”, 88100 Catanzaro, Italy; giovanni.ruotolo@materdominiaou.it; 6 Department of Internal Medicine, and Rare Diseases Centre “C. Frugoni”, University Hospital of Bari, 70120 Bari, Italy

**Keywords:** multidimensional prognostic index, frailty, comprehensive geriatric assessment, agreement

## Abstract

In clinical practice, self-administered and brief tools to promptly identify older people at risk of frailty are required. The Multidimensional Prognostic Index (MPI), derived from the Comprehensive Geriatric Assessment (CGA) seems reliable enough to serve this purpose, but despite the several versions developed over the past 15 years, it lacks a self-administered and brief version. In this study, we aimed to evaluate the agreement between an abbreviated form of the SELFY-MPI (i.e., SELFY-BRIEF-MPI) and the standard version of the MPI. Four Italian hospitals consecutively enrolled outpatients and inpatients >65 years. The sample included 105 participants (mean age = 78.8 years, 53.3% females). Overall, the two versions showed non-statistically significant differences (Standard-MPI 0.42 ± 0.19 vs.. SELFY-BRIEF-MPI 0.41 ± 0.18; *p* = 0.104) and a very strong correlation (R = 0.86, *p* < 0.001). The Bland–Altman Plot revealed that only 5/105 measurements (4.76%) were outside the limits of agreement. The accuracy of the SELFY-BRIEF-MPI in identifying frail people (defined as a Standard-MPI > 0.66) was optimal (area under the curve, AUC = 0.90, *p* < 0.001). To predict multidimensional frailty, a SELFY-BRIEF-MPI score of 0.60 exhibited the greatest sensitivity/specificity ratio. In conclusion, the SELFY-BRIEF-MPI reported a good agreement with the standard version of the MPI, indicating its application in the screening of multidimensional frailty among older people.

## 1. Introduction

Clinicians are constantly looking for tools to gather as much information in as little time as possible in order to conduct quick but effective screenings and detect frail or at-risk individuals early. In fact, frailty, which is a deterioration in the functioning of several physiological systems and an increased susceptibility to stressors [1], increases with age and is linked to a higher risk of negative health outcomes such as hospitalization, institutionalization, falls, and mortality [2]. The prevalence of frailty is estimated as 10.7% in community-dwelling older people [3], and this percentage rises when considering different settings, such as hospitals, with a pooled prevalence of 41.4% [4]. Such variability demonstrates how ageing has a different progression for each individual, and thus a wide range of different dimensions, e.g., functional abilities and polypharmacy, must be taken into account in order to offer a holistic evaluation of this heterogeneous process.

This heterogeneity falls within the concept of multidimensional frailty, which is also related to a higher risk of diseases such as depression [5] and a decrease in quality of life [6]. All these possible consequences highlight the importance of developing and validating tools for prompt detection and the planning of interventions that could likely counteract frailty. In fact, frailty is a reversible condition and early detection, with simple but reliable tools, is highly needed, especially in primary care, to submit patients to more specific assessments and to plan targeted interventions. However, such instruments are not easy to develop as frailty is conceptualized as a multidimensional construct that needs to be evaluated using the Comprehensive Geriatric Assessment (CGA), taking into account all the involved domains.

The CGA is currently considered the gold standard in clinical practice for taking care of older people in hospitals and other settings [7], in order to detect treatable health issues and create a coordinated and personalized care plan to improve overall health as people age. The CGA is a multidimensional, interdisciplinary diagnostic procedure that incorporates a systematic examination of the multiple domains of older people, in particular those who are frail or have complex medical requirements. This assessment is used to collect information on clinical, psycho-social, and biological characteristics to build a personalized care program for the older subject, with important evidence of benefits in medical populations [1]. Moreover, the CGA’s usefulness is deep-rooted, and when it guides tailored interventions, it can result in an enhancement of patients’ functional and cognitive abilities [8,9] and a decrease in medical costs [10,11], hospitalization, and institutionalization [9].

A series of instruments have been developed inspired by the CGA; among them, there is the Multidimensional Prognostic Index (MPI) [12], which is a CGA-based prognostic tool that collects information on functional and cognitive aspects, mobility, nutrition, polypharmacy, comorbidity, and cohabitation status through standardized and validated rating tests [12]. The accuracy of the MPI in predicting short- and long-term mortality and other negative outcomes is confirmed by several multicenter studies, making the MPI a robust and validated tool in the scientific community [13]. From the main instrument, shorter and more practical versions have been developed. Among these, the BRIEF-MPI is a short version of the Standard-MPI that was developed and validated in order to have a screening frailty tool with short administration time to collect information at the patient’s bedside [14]. The BRIEF-MPI has good agreement with the standard version of the MPI, making this tool ideal for the screening of multidimensional frailty in older people [14].

Another version is the SELFY-MPI (Self-Administered Version of the MPI), which focuses on the subjective perception that the patient has of the state of his/her multidimensional conditions [15]. Indeed, self-assessment tools have been extensively employed in epidemiological research, especially as screening tools, because of their usefulness, breadth, speed, and affordability [16]. The SELFY-MPI is based on self-perception of frailty and, different from other self-reported instruments developed for frailty evaluation, it takes into account all the domains assessed by the MPI. These characteristics make it an important tool for the early diagnosis of frailty itself [17].

The literature has affirmed that assessments conducted by self-administration seem quite reliable and appear to have a prognostic role in the mortality of both adults [18] and older people [19], shedding light on the need for the development of new self-administered tools. Moreover, recent research has indicated that socioeconomic and cultural differences can cause short- and long-term disparities in two crucial dimensions for the prevention and management of chronic diseases: healthcare and self-care behaviors. Despite their critical role, some of the available self-administered frailty screening tools rely primarily on physical aspects. Thus, it could be beneficial to also include the assessment of cultural and socioeconomic features, which could affect the general health condition of older people [20]. Given this background, we aimed to evaluate, for the first time, in a population of older subjects, the agreement between an abbreviated form of the SELFY-MPI (i.e., SELFY-BRIEF-MPI) and the standard version of the MPI.

## 2. Materials and Methods

### 2.1. Participants

Participants were older subjects consecutively enrolled at four Italian hospitals chosen on a voluntary basis (Bari, Genova, Catanzaro, and Palermo) from 1st January 2023 to 15th April 2023. Each involved hospital invited older people to participate in the study, recruiting them among the hospitalized patients, outpatients, or both, until 25 subjects per setting were reached. The selection criteria were subjects 65 years and older and able to give written informed consent. Participants who refused to participate were excluded. Before the study was carried out, ethical clearance was obtained on 27th March 2018 in the context of the MULTIPLAT_AGE project (protocol n. 5/2018), and the study was carried out in accordance with the Declaration of Helsinki.

### 2.2. The Multidimensional Prognostic Index (Standard Version)

The Multidimensional Prognostic Index (MPI) is a multidimensional frailty tool able to predict negative outcomes [12].

The MPI comprises eight domains:

1. Activities of Daily Living (ADL [21]): six items to assess the patient’s independence in the management of basic physical needs;
2. Instrumental Activities of Daily Living (IADL [22]): eight items to measure one’s capacity for self-care and home maintenance;
3. Short Portable Mental Status Questionnaire (SPMSQ [23]): ten items for the evaluation of cognitive status;
4. Exton-Smith Scale (ESS [24]): five items to estimate the likelihood of developing pressure sores;
5. Mini-Nutritional Assessment Short Form (MNA-SF [25]): six questions that investigate a person’s nutritional status, the presence of neuropsychological disorders, and exposure to acute stressors;
6. Cumulative Illness Rating Scale (CIRS [26]): fourteen items to identify the existence and severity of concomitant conditions;
7. Number of drugs consumed at home;
8. Cohabitation status (alone, with family, or institutionalized): to indirectly acknowledge psychosocial information.

The raw score for each domain was converted into one of three risk categories associated with a specific score (0 = low, 0.5 = moderate, and 1 = high risk). The sum of all the risk categories was then divided by the number of domains to obtain a standardized continuous value ranging from 0 to 1.00 that could be divided into three categories of risk: MPI-1—mild risk for values below 0.33; MPI-2—moderate risk for MPI values between 0.34 and 0.66; MPI-3—severe risk for MPI values greater than 0.67. The execution of the MPI requires, on average, 15 min [27], and it could be administered by physicians and other healthcare professionals. At the following address, it is possible to download the software for free: https://multiplat-age.it/index.php/en/tools(accessed on 4th August 2023). In Supplementary Table S1, we report how the MPI is built.

### 2.3. The SELFY-BRIEF-MPI

Items from the standard version and the previously validated BRIEF-MPI version [14] were chosen to develop the SELFY-BRIEF-MPI, a short and self-administered version of the MPI that nonetheless assesses the same domains, as follows:

- Three dichotomous answers for the ADL test to evaluate independence in the most relevant areas of feeding, dressing, and continence.
- Three questions about the capacity to make calls, consume medicines, and conduct independent shopping were used to evaluate IADL.
- The Cognitive Change Index (CCI [28]), which uses three yes-or-no questions to assess cognitive status, was used to measure how people perceived their cognitive abilities, particularly their attention, language, and memory skills.
- The mobility domain measures an individual’s capacity to move in and out of a chair or bed, walk, and climb stairs. Each aptitude is classified as either yes or no.
- Two questions about changes in food consumption and weight along with an objective measure (BMI) are used to evaluate the nutritional status.
- The presence of comorbid conditions is assessed in this version through a single question about the number of diseases that require chronic therapies.
- The assessment of the number of medications was unchanged from the standard version.
- Cohabitation status is still tripartite: living alone, with family, or institutionalized, unchanged from the standard version.

Thus, the 53 items of the Standard-MPI were reduced to 18 items, and the answers to the first 5 domains of the SELFY-BRIEF-MPI may only be given in a binary fashion (yes or no). The criteria for the items selection were: (1) prevent repeating the items included in several scales; (2) for the IADL, only choose activities that are most likely to be performed by both genders; (3) the MPI-InChianti template was used for the mobility assessment; (4) the three key points of nutritional status, e.g., body mass index, weight loss, and change in food intake, were included; (5) in general, the hierarchy in the progression of loss of functions guided our decisions. The selected items are, therefore, the same as the BRIEF-MPI [14], except for the cognitive status assessment, which, as it is self-administered, focuses on a subjective evaluation of memory impairments. Concerning the cognitive domain, the CCI was chosen because it showed high accordance between the self- and informant administration, likely allowing for only a small loss of information due to self-reporting and evaluating one’s own cognitive abilities. Furthermore, the literature highlights the predictive role of subjective memory impairments in dementia [29]. The CCI was composed of 20 items assessing 3 different cognitive domains (memory, executive function, and language); for this reason, we selected 1 item for each domain.

In conclusion, the final score of the SELFY-BRIEF-MPI is calculated in a manner similar to that of the Standard-MPI: a risk category can be derived for each domain, which is added up and then divided by the total number of domains completed. The result can be grouped into three MPI risk categories with the same cut-off scores, ranging from 0 (lowest risk) to 1.00 (highest risk). The duration of the administration is comparable to that of the BRIEF-MPI version, which typically lasts about 5 min. In Supplementary Table S2, we report how the SELFY-BRIEF-MPI is built.

### 2.4. Administration Procedure

After giving their written consent, patients were assessed through the Standard-MPI, which was administered by the physicians visiting them in hospitals or outpatient clinics. On the same day, the SELFY-BRIEF-MPI was completed, which was self-administered by all the participants. Along with the two MPI versions, further sociodemographic information was collected from the physicians (e.g., cause of hospitalization). For the hospitalized participants, both evaluations were carried out after the acute events and following the stabilization of their health conditions, to reduce the possible influencing impact of the episode that caused hospitalization.

### 2.5. Statistical Analysis

Since the literature suggests that a difference in the mean MPI is clinically significant for a value of 0.03 or more, we determined that a sample size of at least 100 subjects could be sufficient for detecting a difference of less than 0.03 between the SELFY-BRIEF and the standard form of the MPI, hypothesizing a type I error of 5% and a type II error of 20%.

The means (Ms) and standard deviations (SDs) for continuous variables and frequencies and percentages for categorical variables were used to summarize the sociodemographic and clinical features of individuals. Each variable was tested for normality distribution using the Shapiro-Wilk test. As all the dimensions were non-normally distributed, non-parametrical analyses were employed. Three distinct techniques were employed to evaluate the agreement between the SELFY-BRIEF version and the standard one: 1. Comparison between the MPI index and its domains (category of risk) through Wilcoxon’s signed ranks. 2. Correlation analysis, using Spearman’s R; and 3. The Bland–Altman plot (BAP). This last analysis consists of a graphic depiction of the difference and the mean of the measurements and an objective evaluation of the agreements, reporting the number of observations that fall within the 95% confidence intervals (CIs) [30,31]. The area under the curve (AUC) was then examined to gauge how well the SELFY-BRIEF-MPI predicted the existence of multidimensional frailty, defined as a value of the Standard-MPI > 0.66, i.e., the two predicted conditions were frail vs. robust, and belonging to one of them was defined by reporting a score under 0.66 (robust people) or below it (frail people) on the Standard-MPI [32]. The best cut-off point that maximized the instrument’s sensitivity and specificity was selected using Youden’s index [33]. All two-tailed statistical tests were deemed statistically significant at a *p*-value of 0.05 or less. STATA (version 14.0 for Windows) and SPSS (version 21.0 for Mac) were both used for all the analyses.

## 3. Results

In each center, 25 older people were invited to participate, except for Genoa, where the target number was 50 (25 among the hospitalized and 25 outpatients). Overall, the participation rate was 84% (80% for Bari and Catanzaro, 96% for Palermo, and 82% for Genoa). Participants and those who refused to take part in the study did not differ in basic characteristics.

The study included 105 participants: 20 of them were from Bari, 20 from Catanzaro, 24 from Palermo, and 41 from Genoa. The health status of the study sample was quite heterogeneous, as the most frequent diseases were cardiovascular (n = 21) and cognitive impairments (n = 21). The other participants reported suffering from respiratory disease (n = 19) and infectious pathologies (n = 11). Less likely diseases were psychological disorders (n = 6), hepatological issues (n = 6), urinary (n = 4) and gastrointestinal disorders (n = 3), endocrine-metabolic diseases (n = 4), skeletal-muscle issues (n = 4), hematological disorders (n = 2), neurological (n = 2) and oncological pathologies (n = 2). The main characteristics of the participants divided by each recruitment center are included in Supplementary Table S3.

The mean age was 78.8 ± 7.0 years (range 65–99), and 53.3% of participants were females. Fifty of them were outpatients who went to the hospital for routine visits, whilst 55 were hospitalized. The Standard-MPI mean was 0.42 ± 0.19 with 33 participants in MPI-1 (31.5%), 62 in MPI-2 (59%), and 10 in MPI-3 (9.5%). The characteristics of the sample as determined by the MPI standard version are displayed in Table 1.

Table 2 shows the comparison between the Standard-MPI and the SELFY-BRIEF-MPI for each domain and the overall score. Overall, the two versions were not statistically different (Mean Standard-MPI 0.42 ± 0.19 vs. SELFY-BRIEF-MPI 0.41 ± 0.18; *p* = 0.104). Similarly, neither ADL (*p* = 1.000) nor CIRS (*p* = 0.275) significantly differed. Furthermore, cohabitation status and number of drugs were identical (both *p* = 1.000). Statistically significant differences in the domains were found in IADL (*p* = 0.007), MNA-SF (*p* < 0.001), ESS-MOB (*p* = 0.029), and SPMSQ-CCI (*p* < 0.001).

**Figure 1:**
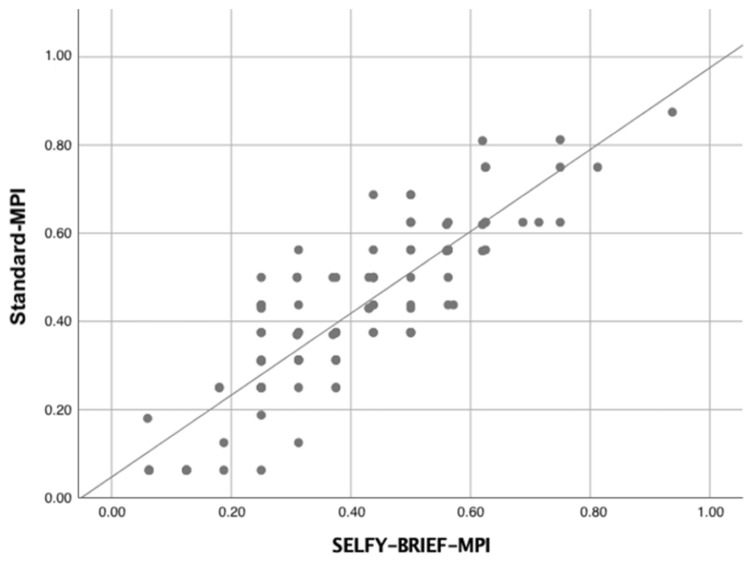
Linear correlation between the Standard-MPI and the SELFY-BRIEF-MPI.

The Standard-MPI version and the SELFY-BRIEF-MPI displayed a very strong correlation (R = 0.86; *p* < 0.001) (Figure 1).

**Figure 2:**
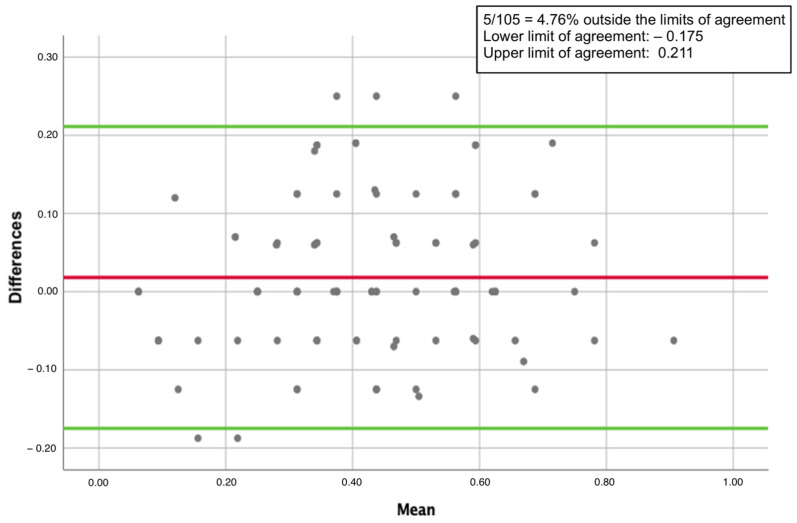
The Bland–Altman plot for the agreement between the Standard-MPI and the SELFY-BRIEF-MPI. The red line indicates the mean of the differences between the measurements obtained with the two instruments. The green lines represents the upper and lower limits of agreement.

Additionally, the BAP revealed that only 5 participants among the 105 included (4.76%) were outside the limits of agreement (Figure 2).

**Figure 3:**
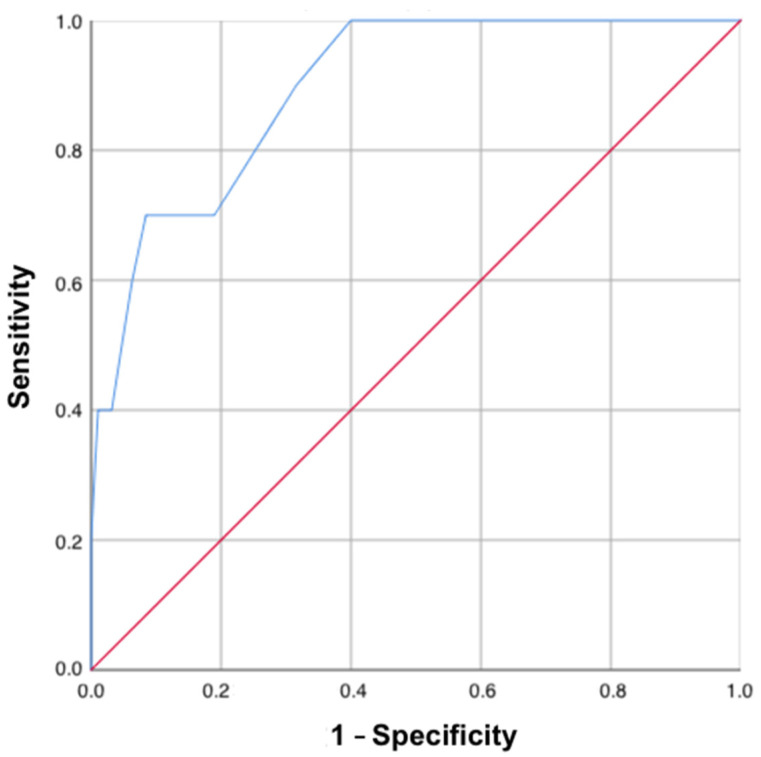
ROC curve (in blue) for the accuracy of the SELFY-BRIEF-MPI in determining frailty using the 0.66 cut-off of the Standard-MPI version. The red line instead represents a classifier that performs a random assigning.

Finally, to test the accuracy of the SELFY-BRIEF-MPI in determining the presence of multidimensional frailty, we calculated the area under the curve (AUC) of the SELFY-BRIEF-MPI to identify older subjects with a Standard-MPI score above 0.66, i.e., frail subjects. As shown in Figure 3, the receiver operating characteristic (ROC) curve of the SELFY-BRIEF-MPI was 0.90 (*p* < 0.001), suggesting that the SELFY-BRIEF-MPI is capable of identifying 90% of subjects with multidimensional frailty, as determined by the standard version of the MPI (MPI score > 0.66). The best sensitivity/specificity ratio in identifying multidimensional frailty using the SELFY-BRIEF-MPI score was observed at a SELFY-BRIEF-MPI value of 0.60, exhibiting a sensitivity of 70% and a specificity of 91.6%. Furthermore, the SELFY-BRIEF-MPI score that showed the best sensitivity (100% value) was 0.43, whilst the most specificity (100% value) was at 0.78.

## 4. Discussion

This work, including 105 older in- and outpatients from 4 different centers in Italy, demonstrated that the abbreviated form of a self-administered form of the MPI, i.e., the SELFY-BRIEF-MPI, had a good agreement with the standard version of the MPI, which represents the gold standard in clinical practice and research [34]. This study shows that the SELFY-BRIEF-MPI could be able to identify frail older patients, being useful in estimating the needs of older people. The SELFY-MPI and the BRIEF-MPI should be used in frailty screening, as shown in previous studies [12,15]. Similarly, the SELFY-BRIEF-MPI demonstrated good accuracy since it predicts around 90% of multidimensional frailty. It is evident how the use of screening tests such as the SELFY-MPI or the BRIEF-MPI can be useful in clinical practice to intercept older people who need multidimensional and multidisciplinary interventions.

In recent years, there has been a growing interest in self-assessment tools, dictated above all by the need to obtain very important information in a short time, such as that of the estimation of frailty [17]. Frailty, in fact, is potentially reversible if detected early and appropriately treated [35]. Therefore, a brief and self-administered version of the MPI could be a useful instrument to assess and identify older subjects who are frail and/or are at high risk for frailty. This detection could help physicians in submitting patients to specialistic examinations and planning the most useful tailored multidimensional interventions. This study demonstrates that the SELFY-BRIEF-MPI may give extremely accurate and valid results to identify the needs of older subjects in terms of multidimensional impairment. The results are comparable to those obtained by the standard version of the MPI, which requires the intervention of healthcare professionals and takes almost three times as long as the SELFY-BRIEF-MPI.

Remarkably, no significant differences in mean MPI between the standard and SELFY-BRIEF-MPI versions emerged. However, significant differences are present in nutrition, mobility, instrumental activities of daily living, and cognitive domains. We can justify these findings since the screening tests for these domains are somewhat different from the standard versions. For example, the questions used in the screening version, i.e., in the SELFY-BRIEF-MPI version of the nutritional domain, include only nutritional items, whilst in the MNA, other relevant aspects for nutrition (e.g., the presence of dementia or stress) are included. Similarly, for the cognitive domain, we only asked for subjective cognitive complaints. Finally, one possible reason for the differences in the instrumental activities of daily living could be that some gender-related activities were excluded from the SELFY-BRIEF-MPI and thus did not affect the risk category. In the standard version, instead, items concerning household activity, which is traditionally more common among older women than older men, could cause a worse IADL score than the new version.

The findings of this study must be interpreted within its limitations. First, the cohort enrolled was relatively small, consisting of 105 participants. However, in future studies, we plan to include a bigger sample size of older subjects recruited in different settings and contexts. A greater number of participants could also allow us to stratify the sample and evaluate the possible effects of gender or age on the differences found among the MPI domains of the two versions. Second, the Standard-MPI mean was 0.42 ± 0.19 with 33 participants in MPI-1 (31.5%), 62 in MPI-2 (59%), and only 10 in MPI-3 (9.5%). These findings suggest the need to further validate the SELFY-BRIEF-MPI in different settings, including long-term care facilities, in order to test this screening frailty tool in a frailer population. Third, some domains are significantly different between the standard and brief versions of the MPI: in particular, the SPMSQ and MNA seem to be underestimated by the SELFY-BRIEF-MPI, compared to the standard version, suggesting that more research is needed. Finally, the majority of people included were from a hospital setting (in- or outpatient), whilst the application of the SELFY-BRIEF-MPI could be more useful in other settings, such as the emergency department and/or in primary care.

## 5. Conclusions

In conclusion, the SELFY-BRIEF-MPI has a good agreement with the standard version of the MPI. For this reason, this tool could be used for identifying older people who are potentially frail earlier as well as the domains that could be the target of personalized interventions. Future studies are needed in larger populations to confirm these findings.

## Figures and Tables

**Table 1:** Baseline characteristics of the participants included in the study and their Standard-MPI scores (total and for each domain).

Parameter	Mean	Standard Deviation	Range
**Age**	78.75	7.03	65–99
**Standard-MPI**	0.42	0.19	0.06–0.88
**ADL**	3.70	1.87	0–6
**IADL**	3.91	2.57	0–8
**SPMSQ**	2.17	2.27	0–10
**ESS**	12.36	6.36	0–20
**MNA-SF**	7.38	4.63	0–14
**CIRS**	4.49	2.62	0–13
**N. OF DRUGS**	6.46	3.34	1–14
**Cohabitation status:** Living alone 27 (25.7%)			
**Cause of hospitalization/visiting outpatients’ clinics:** - Cardiovascular diseases: 21 (20.0%). - Cognitive impairments: 21 (20.0%). - Respiratory diseases: 19 (18.1%). - Infectious pathologies: 11 (10.5%). - Psychological disorders: 6 (5.7%). - Hepatological issues: 6 (5.7%). - Urinary disorders: 4 (3.8%). - Gastrointestinal disorders: 3 (2.9%). - Endocrine-metabolic diseases: 4 (3.8%). - Skeletal-muscle diseases: 4 (3.8%). - Hematological disorders: 2 (1.9%). - Neurological disorders: 2 (1.9%). - Oncological pathologies: 2 (1.9%).			

Abbreviations: MPI, Multidimensional Prognostic Index; ADL, Activities of Daily Living; IADL, Instrumental Activities of Daily Living; SPMSQ, Short Portable Mental State Questionnaire; ESS, Exton-Smith Scale; MNA-SF, Mini-Nutritional Assessment–Short Form; CIRS, Cumulative Illness Rating Scale.

**Table 2:** Comparison between the Standard-MPI and the SELFY-BRIEF-MPI.

MPI’s Domains		Standard-MPI	SELFY-BRIEF-MPI	*p*-Value
**MPI index** (n = 105)		0.42 (0.19)	0.41 (0.18)	0.104
**ADL** (n = 105)	Low (n, %)	62 (59%)	58 (55.2%)	1.000
	Medium (n, %)	19 (18.1%)	27 (25.7%)	
	High (n, %)	24 (22.9%)	20 (19%)	
**IADL** (n = 105)	Low (n, %)	49 (46.7%)	50 (47.6%)	0.007
	Medium (n, %)	16 (15.2%)	29 (27.6%)	
	High (n, %)	40 (38.1%)	26 (24.8%)	
**MNA-SF** (n = 105)	Low (n, %)	29 (27.6%)	66 (62.9%)	<0.001
	Medium (n, %)	48 (45.7%)	34 (32.4%)	
	High (n, %)	28 (26.7%)	5 (4.8%)	
**CIRS-CI** (Standard-MPI n = 104; SELFY-BRIEF-MPI n = 105)	Low (n, %)	5 (4.8%)	0 (0%)	0.279
	Medium (n, %)	16 (15.4%)	32 (30.5%)	
	High (n, %)	83 (79.8%)	73 (69.5%)	
**ESS-MOB** (n = 105)	Low (n, %)	62 (59%)	79 (75.2%)	0.029
	Medium (n, %)	34 (32.4%)	16 (15.2%)	
	High (n, %)	9 (8.6%)	10 (9.5%)	
**SPMSQ** (n = 105) **CCI** (n = 104)	Low (n, %)	74 (70.5%)	54 (51.9%)	<0.001
	Medium (n, %)	23 (21.9%)	24 (23.1%)	
	High (n, %)	8 (7.6%)	26 (25%)	
**Cohabitation status** (n = 105)	Low (n, %)	76 (72.4%)	76 (72.4%)	1.000
	Medium (n, %)	2 (1.9%)	2 (1.9%)	
	High (n, %)	27 (25.7%)	27 (25.7%)	
**Number of drugs** (n = 104)	Low (n, %)	23 (22.1%)	23 (22.1%)	1.000
	Medium (n, %)	27 (26%)	27 (26%)	
	High (n, %)	54 (51.9%)	54 (51.9%)	

Abbreviations: MPI, Multidimensional Prognostic Index; ADL, Activities of Daily Living; IADL, Instrumental Activities of Daily Living; SPMSQ, Short Portable Mental State Questionnaire; CCI, Cognitive Change Index; ESS, Exton-Smith Scale; MOB, Mobility Domain; MNA-SF, Mini-Nutritional Assessment–Short Form; CIRS, Cumulative Illness Rating Scale.

## Data Availability

Data are available upon request to the corresponding author.

## Supplementary Materials

The following supporting information can be downloaded at: https://www.mdpi.com/article/10.3390/jcm12186026/s1, Table S1: Standard-MPI; Table S2: The SELFY-BRIEF-MPI; Table S3: Basic features of the participants divided by each recruitment center.
