# Supplementary material for: Validation of a Brief Form of the Self-Administered Multidimensional Prognostic Index: The SELFY-BRIEF-MPI Project
Source: J Clin Med. 2023 Sep 18;12(18):6026. doi: 10.3390/jcm12186026 (PMC10531940; doi:10.3390/jcm12186026)
Supplement: Supplementary file 1 [file jcm-12-06026-s001.zip › jcm-2572255-supplementary.pdf]

Supplementary table S1. Standard-MPI

1. ACTIVITIES OF DAILY LIVING (ADL) *

Is the patient able to perform the following activities completely independently?

A) **BATHING** (either sponge bath, tub bath, or shower)	
- YES	1
- NO	0
B) **DRESSING**	
- YES	1
- NO	0
C) **TOILETING** (going to the "toilet room" for bowel and urine elimination, cleaning self after elimination, and arranging clothes)	
- YES	1
- NO	0
D) **TRANSFER**	
- YES	1
- NO	0
E) **CONTINENCE**	
- YES	1
- NO	0
F) **FEEDING**	
- YES	1
- NO	0

TOTAL __________

2. INSTRUMENTAL ACTIVITIES OF DAILY LIVING SCALE (IADL)*

Are you able to perform the following activities completely independently?

A) **ABILITY TO USE TELEPHONE (at least answer the phone)**	
Yes	1
No	0
B) **SHOPPING**	
Yes	1
No	0
C) **FOOD PREPARATION**	
Yes	1
No	0
D) **HOUSEKEEPING**	
Yes	1
No	0
E) **LAUNDRY**	
Yes	1
No	0
F) **MOVE INDEPENDENTLY THROUGH PUBLIC TRANSPORT / OWN CAR**	
Yes	1
No	0
G) **RESPOSIBILITY FOR OWN MEDICATIONS**	
Yes	1
No	0
H) **ABILITY TO HANDLE FINANCES housekeeping, paying rent and other expenses, going to the bank/post office)**	
Yes	1
No	0
**TOTAL**	

3.b EXTON-SMITH SCALE (ESS) *

(evaluation of pressure sores risk)

**General Condition**		**Incontinence**	
Bad	1	Doubly incontinent	1
Poor	2	Usually of urine	2
Fair	3	Occasional	3
Good	4	Not	4
**Mental State**		**Mobility in Bed**	
Stuporosous	1	Immobile	1
Confused	2	Very limited	2
Apathetic	3	Slightly limited	3
Alert	4	Full	4
**Activity**			
Lying down		**TOTAL** __________	
Sitting		*Score 16-20: minimum risk*	
Walk with help		*Score 10-15: medium risk*	
Walk		*Score 5-9: high risk*	

4. SHORT PORTABLE MENTAL STATUS QUESTIONNAIRE (SPMSQ)

	Correct	Wrong
What is the date today? (Correct only when the month, date, and year are all correct)	0	1
What day of the week is it?	0	1
What is the name of this place? (Correct if any of the description of the location is given)	0	1
What is your street address?	0	1
How old are you?	0	1
When were you born?	0	1
Who is the president (or the Pope) now? (Requires only the correct last name)	0	1
Who was president (or the Pope) just before him?	0	1
What was your mother's maiden name?	0	1
Subtract 3 from 20 and keep subtracting 3 from each new number at least for 3 times (The entire series must be performed correctly to be scored as correct)	0	1

TOTAL: ____________________

5. MINI NUTRITIONAL ASSESSMENT Short Form (MNA - SF)

Weight:________________kg	Height:________________cm

Screening	
**A Does the patient present with a loss of appetite? Have he/she eaten less in the past 3 months? (loss of appetite, digestive problems, difficulty chewing or swallowing)**<br><br>2= No, his/her food intake hasn't decreased<br>1 = Yes, his/her food intake decreased moderately<br><br>0= Yes, his/her food intake decreased severely	
**B Weight loss (<3 months)**<br>0 = wieght loss> 3 kg<br>1 = he/she does not know<br>2 = weight loss between 1 and 3 kg<br>3 = no weight loss	
**C Mobility**<br>0 = bed or chair bound<br>1 = able to get out of bed/chair but does not go out<br>2 = goes out	
**D Has suffered psychological stress or acute disease in the past 3 months**<br>0 = yes<br>2 = no	
**E Neuropsychological problems**<br>0 = severe dementia or depression<br>1 = mild dementia<br>2 = no psychological problems	
**F1 Body Mass Index (BMI) =** weight in kg / (height in m)^2<br>0 = BMI<19<br>1 = 19 ≤ BMI < 21<br>2 = 21 ≤ BMI < 23<br>3 = BMI ≥ 23	
IF BMI IS NOT AVAILABLE, REPLACE QUESTION F1 WITH QUESTION F2.<br>DO NOT ANSWER QUESTION F2 IF QUESTION F1 HAS ALREADY BEEN COMPLETED.	
**F2 Calf Circumference (CC in cm)**<br>0 = CP less than 31<br>3 = CP 31 or superior	

**Screening Evaluation**<br>(maximum 14 points)	
**12- 14 punti**	**Well-nourished**
**8-11 punti**	**At risk of malnutrition**
**0-7 punti**	**Malnourished**

6. CUMULATIVE ILLNESS RATING SCALE (C.I.R.S.)

	NONE	MILD	MODERATE	SEVERE	EXTREMELY SEVERE
**1.** Cardiac (heart only)	1	2	3	4	5
**2.** Hypertension (rating is based on severity)	1	2	3	4	5
**3.** Vascular (arteries, veins, lymphatics)	1	2	3	4	5
**4.** Respiratory (lungs, bronchi, trachea)	1	2	3	4	5
**5.** EENT (eye, ear, nose, throat, larynx)	1	2	3	4	5
**6.** Upper GI (esophagus, stomach, duodenum, biliary and pancreatic trees)	1	2	3	4	5
**7.** Lower GI (intestines, hernias)	1	2	3	4	5
**8.** Hepatic (liver only)	1	2	3	4	5
**9.** Renal (kidneys only)	1	2	3	4	5
**10**. Other GU (ureters, bladder, urethra, prostate, genitals)	1	2	3	4	5
**11**. Musculo-skeletal-integumentary (muscles, bone, skin)	1	2	3	4	5
**12.** Neurological (brain, spinal cord, nerves)	1	2	3	4	5
**13.** Endocrine-metabolic (including diabetes, hyperlipidemia, infections, toxicity)	1	2	3	4	5
**14.** Psychiatric (dementia, depression, anxiety, agitation, psychosis)	1	2	3	4	5

**ILLNESS SEVERITY SCORE (CIRS-IS)**<br>mean of all single item<br>(excluded the psychiatric item)	**COMORBIDITY INDEX (CIRS-CI)**<br>number of items with a score<br>of 3 or greater (excluded the psychiatric item)

7. Number of drugs used: ________________________

8. Co-habitation status:

Does the patient live:

☐ Alone ☐ With relatives/nurse ☐ In institution

MPI - Multidimensional Prognostic Index			
	Punteggio assegnato a ogni dominio		
	**Low (Value = 0)**	**Mild (Value = 0.5)**	**High (Value = 1)**
**1. ADL**	6-5	4-3	2-0
**2. IADL**	8-6	5-4	3-0
**3. BARTHEL MOB**	3-2	1	0
**4. SPMSQ**	0-3	4-7	8-10
**5. MNA-SF**	12-14	8-11	0-7
**6. CIRS**	0	1-2	≥3
**7. Number of drugs**	0-3	4-6	≥7
**8. Social status**	Lives with family	Institutionalized	Living alone
Add up the scores assigned to each domain, and then divide the sum by 8		**TOTAL SCORE MPI OUTPATIENT VERSION**	

Legend:

RISK	Mild (MPI 1)	Moderate (MPI 2)	.Severe (MPI 3)
**RANGE**	**0.00 - 0.33**	**0.34-0.66**	**0.67-1.0**

Supplementary Table S2. SELFY-BRIEF-MPI

**SELFY-BRIEF-MPI (Multidimensional Prognostic Index)**				
**Domain**	**Source**	**Item**	**Score**	**Summed score by domain**
Basic Activities of Daily Living	ADL	I am able to eat on my own, without needing any assistance	0= No<br>1= Yes	________
		I am able to get dressed by myself without needing any assistance	0= No<br>1= Yes	
		I have complete bowel and bladder control	0= No<br>1= Yes	
Instrumental Activities of Daily Living	IADL	I can use the telephone by myself	0= No<br>1= Yes	________
		I take my medication by myself in the correct dosage and at the correct time	0= No<br>1= Yes	
		I can take care of grocery shopping by myself, without needing any assistance	0= No<br>1= Yes	
Mobility	MPI-InChianti	I can get in and out of bed or chair by myself without needing any assistance	0= No<br>1= Yes	________
		I am able to walk by myself for more than 50 meters on a flat ground (even using walking aids such as walking stick, walker, etc..)	0= No<br>1= Yes	
		I am able to walk up and down stair without needing any assistance	0= No<br>1= Yes	
Cognitive status	CCI	I find it more difficult to remember recent events than I used to	0= No<br>1= Yes	________
		I find it more difficult to complete a task than I used to	0= No<br>1= Yes	
		I find it more difficult to follow a conversation than I used to	0= No<br>1= Yes	
Nutrition	MNA mod.	Body Mass Index (BMI) =<br>Weight (Kg)<br>---------------------- = ________<br>Height ^{2} (in metres)<br>My total BMI value is between 21 and 29,9	0= Yes<br>1= No	________
		In the last 3 months, my food intake decreased severely due to a loss of appetite, digestive problems, chewing or swallowing difficulties	0= No<br>1= Yes	

		In the last 3 months, I lost more than 3 Kg (6.6 Ibs)	0= No<br>1= Yes	
Comorbidity	CIRS<br>Selfy-MPI	Please, indicate the number of health problems for which you need medication (extended over time)	———(from 0 to 13)	//
N. of drugs	Selfy-MPI	How many different medications do you take a day?	———	//
Co-habitation status	Selfy-MPI	What is your household composition?	ϒ I live with family / husband / wife / caregiver<br>ϒ I live in a residential home for elderly people<br>ϒ I live alone	//

SELFY-BRIEF-MPI : SCORING

SELFY-BRIEF-MPI (Multidimensional Prognostic Index)				
	Score assigned to each domain			Value of the corresponding column<br><br>**(0- 0.5- 1)**
	Low<br><br>**(Value = 0)**	Moderate<br><br>**(Value = 0.5)**	High<br><br>**(Value = 1)**	
**ADL**	3	2	1 or 0	
**IADL**	3	2	1or 0	
**MOBILITY**	3-2	1	0	
**Cognitive status**	0-1	2	3	
**Nutritional status**	0-1	2	3	
**Comorbidity**	0	1-2	≥3	
**N. of drugs**	0-3	4-6	≥7	
**Co-habitation status**	With family / husband / wife / caregiver	In residential home	Alone	
Add up the scores obtained in the last column on the right				Tot. ________
Divide the total score by 8			**Final score**<br><br>**SELFY-Brief-MPI**	________: 8 =<br><br>_________

Supplementary Table S3. Basic features of the participants divided by each recruitment center.

Center	Gender (n. of males)	Enrollment Setting	Age (M ±SD)	Standard-MPI (M ±SD)	SELFY-BRIEF-MPI (M ±SD)
**Bari (n=20)**	13	4 outpatients and 16 hospitalized	80.3 ± 9.0	0.39±0.23	0.38±0.18
**Catanzaro (n=20)**	12	20 outpatients	73.9±3.6	0.35±0.16	0.38±0.17
**Genoa (n=41)**	12	26 outpatients and 15 hospitalized	81.5±6.4	0.47±0.20	0.45±0.19
**Palermo (n=24)**	12	24 hospitalized	76.9±5.8	0.44±0.14	0.37±0.16
